# The Time Course of Gene Expression during Reactive Gliosis in the Optic Nerve

**DOI:** 10.1371/journal.pone.0067094

**Published:** 2013-06-27

**Authors:** Juan Qu, Tatjana C. Jakobs

**Affiliations:** Massachusetts Eye and Ear Infirmary, Department of Ophthalmology, Harvard Medical School, Boston, Massachusetts, United States of America; Hertie Institute for Clinical Brain Research, University of Tuebingen., Germany

## Abstract

Reactive gliosis is a complex process that involves changes in gene expression and morphological remodeling. The mouse optic nerve, where astrocytes, microglia and oligodendrocytes interact with retinal ganglion cell axons and each other, is a particularly suitable model for studying the molecular mechanisms of reactive gliosis. We triggered gliosis at the mouse optic nerve head by retro orbital nerve crush. We followed the expression profiles of 14,000 genes from 1 day to 3 months, as the optic nerve formed a glial scar. The transcriptome showed profound changes. These were greatest shortly after injury; the numbers of differentially regulated genes then dropped, returning nearly to resting levels by 3 months. Different genes were modulated with very different time courses, and functionally distinct groups of genes responded in partially overlapping waves. These correspond roughly to two quick waves of inflammation and cell proliferation, a slow wave of tissue remodeling and debris removal, and a final stationary phase that primarily reflects permanent structural changes in the axons. Responses from astrocytes, microglia and oligodendrocytes were distinctively different, both molecularly and morphologically. Comparisons to other models of brain injury and to glaucoma indicated that the glial responses depended on both the tissue and the injury. Attempts to modulate glial function after axonal injuries should consider different mechanistic targets at different times following the insult.

## Introduction

Damage to central nervous tissue leads to reactive gliosis, often resulting eventually in a glial scar. Originally described as a simply hypertrophy of astrocytes and increased expression of glial fibrillary acidic protein (GFAP), astrocyte reactivity is now known to be a complex process that involves changes in gene expression and morphological changes in astrocyte architecture [1], [2]. More is known about the reactivity of protoplasmic astrocytes than about white matter (fibrous) astrocytes. Protoplasmic astrocytes occupy non-overlapping spatial domains [3]. They lose their domain organization upon some injuries, but maintain it upon others [4], [5]. White matter astrocytes start out with a large degree of spatial overlap and respond to injury in a biphasic manner. In a first phase, they retract processes and reduce spatial coverage, and in a second phase they reextend long processes [6]. These examples show that astrocyte reactivity is not a generic process but depends on the type of injury and the localization of the cells in the brain. On the level of gene expression, it has also been demonstrated that astrocyte reactivity is a heterogeneous process. A comparison between cerebral ischemia and LPS-induced neuroinflammation showed that a subset of differentially regulated genes were identical, but many were unique to the condition studied [7].

To better understand the molecular mechanisms in reactive gliosis, we chose Affymetrix GeneChip analysis of optic nerve head (ONH) tissue after crush injury. In the ONH, astrocytes form the direct neighborhood of the unmyelinated axons, which they ensheath and organize into bundles [8], [9]. In addition to astrocytes and axons, the ONH contains microglia [10] and, in the myelination transition zone, oligodendrocytes. The ONH has been shown to be the initial site of insult to retinal ganglion cell (RGC) axons in glaucoma [11], [12], [13], [14], [15], [16], and gene expression changes in that region have been investigated in models of ocular hypertension [17] and glaucoma [18]. We have extended these studies to a timeline of gene expression changes from 1 day to 3 months after injury. We were interested in whether the morphological recovery of astrocytes we reported previously [6] is accompanied by a return to baseline of the gene expression profile, and whether the affected genes follow a parallel time course. We find that optic nerve crush is followed by a strong and immediate response of genes involved in immune response and inflammation, and genes involved in the regulation of cell proliferation. Other genes have slower time courses. Most of these changes resolve by 3 weeks after crush. Only a small group of genes remained differentially regulated until 3 months after injury. These genes were mostly involved in debris clearance and tissue repair.

## Materials and Methods

### Animals

All animal procedures were approved by the Research Animal Care Committee of the Massachusetts Eye and Ear Infirmary and in accordance with the ARVO Statement for the Use of Animals in Ophthalmic and Vision Research. C57BL/6 mice, hGFAPpr-tomato mice, and D2.hGFAPpr-EGFP mice were used in this study. C57BL/6 mice were purchased from Charles River Laboratories. hGFAPpr-tomato mice were created by crossing the strain B6;129S6-Gt(Rosa)^26Sortm9(CAG-tdTomato)Hze^/J (Jackson Laboratories) with a mouse line that expresses cre recombinase under the control of the human GFAP promoter (generous gift from Dr. David Paul at Harvard Medical School). This results in astrocytes in the brain and the optic nerve expressing red fluorescent protein. D2.hGFAPpr-EGFP mice were created by backcrossing the line hGFAPpr-EGFP [19] that shows expression of GFP in many, but not all, astrocytes into the DBA/2J strain of mice (Jackson Laboratories) which develops glaucoma. Offspring from crosses for at least 6 generations were used.

### Optic Nerve Crush

**Figure 1:**
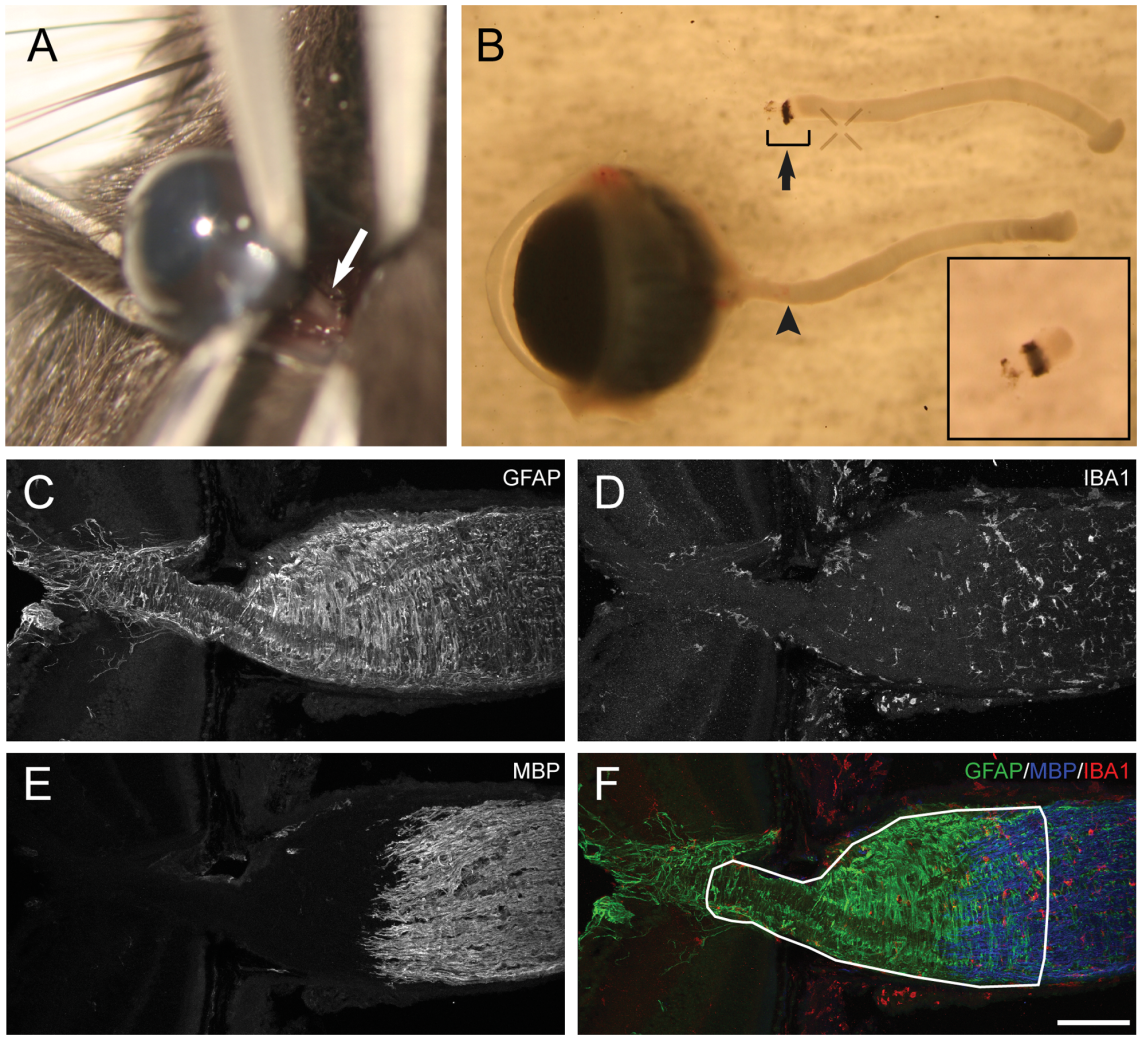
The anatomy of the ONH. A. The optic nerve of the left eye was exposed superiorly by blunt dissection of the conjunctiva, and crushed at about 1 mm behind the globe. The white arrow points at the optic nerve. B. The isolated eye, optic nerve and ONH. The black arrowhead points at the crush site. The bracket and the black arrow indicate the focus of this study – the ONH and the myelination transition zone. A magnified view of this region is shown in the lower right insert. Note the transition from translucent ONH to opaque myelination transition zone. Also note that some of the sclera was left intentionally on this ONH for demonstration purpose, but it was further removed from all the ONHs used in RNA extraction. The upper left insert shows the schematic view of the optic nerve crush. The region circled with the red line is displayed in C–F. C–F. The distribution of the glial cells in the normal ONH. Immunohistochemistry against cell markers for astrocyte (GFAP), microglia (IBA1) and oligodendrocyte (MBP) was performed in longitudinal sections. The retina is on the left side in each image. The white circle in F outlines the tissue used in the gene expression assays. Scale bar, 100 microns.

Optic nerve crush was performed in C57BL/6 mice and GFAPpr-tomato mice. All the mice were female and were 6–8 week old at the time of surgery. Mice were anesthetized by intraperitoneal injection of Ketamine and Xylazine (100 mg/kg and 20 mg/kg body weight). A drop of 0.5% proparacaine was applied to the eye for local anesthesia. A small incision was made in the conjunctiva at the superior pole of the eye. Through this incision, a blunt dissection of the conjunctiva was made with forceps towards the back of the eye to expose the retrobulbar optic nerve (Fig. 1A). Care was taken to avoid any damage to the ocular muscles or to the venous sinuses. The optic nerve was crushed with jeweler’s forceps for 10 seconds at 1 mm behind the globe under direct visual control, avoiding the vascular supply to the eye. After the surgery, bacitracin ointment was applied to avoid infection and the animal recovered on a warming pad. Only the optic nerve of the left eye was crushed, the right eye served as negative control. In case of any complication, the mouse was immediately euthanized and excluded from the study. In order to identify proliferating cells, a group of mice received daily intraperitoneal injections of 5-bromo-2′-deoxyuridine (BrdU, 100 mg/kg body weight, Sigma) for up to 7 days after the crush.

### Tissue Harvest

Mice were euthanized with carbon dioxide followed by cervical dislocation. The skull and the brain were removed, and the eye together with the optic nerve was carefully dissected out without imposing any mechanical stress or damage to the tissue (Fig. 1B). Further processing depended on the experiments and is described as follows.

### Retinal Cell Counts

The retinas were flat-mounted on a nitrocellulose filter paper (Millipore), fixed with 4% paraformaldehyde in PBS for 1 hour, stained with To-Pro-1 (Invitrogen) 1∶1000 in PBS for 15 minutes, and mounted with Vectashield mounting medium (Vector Laboratories). In fixed tissues To-Pro-1 acts as a universal nuclear stain without preference for any cell type. An Olympus BX51 microscope with an Olympus DP70 camera was used to image the retina. Four mid-periphery regions (each region was 232 microns by 232 microns in size) in the ganglion cell layer were imaged in the four quadrants of the retina. The number of nuclei in each region was manually counted in ImageJ. The mean of the four regions was taken as the cell count of the retina.

### Immunohistochemistry

The cornea was cut off along the limbus and the lens and vitreous were removed. The remaining eye cup with the optic nerve was fixed in 4% paraformaldehyde in PBS for 2 hour, protected with 30% sucrose in PBS at 4°C overnight and embedded in O.C.T. compound (Tissue-Tek). A Leica cryostat was used to section the optic nerve either longitudinally or transversely at 14-micron thickness.

The tissue sections were blocked with 4% normal donkey serum (Jackson ImmunoResearch) and 0.1% Triton X 100 in PBS for 1 hour, incubated with primary antibodies overnight and secondary antibodies for 3 hours in the same blocking solution, and mounted with Vectashield mounting medium. The primary antibodies used in this study were: chicken anti GFAP (1∶1000, Abcam), mouse anti SMI32 (1∶1000, Covance), rabbit anti IBA1 (1∶200, Wako), rabbit anti AQP4 (1∶200, Alomone), rabbit anti TNC (1∶100, Millipore), rat anti MBP (1∶200, Abcam), mouse anti THBS1 (1∶100, NeoMarkers), and mouse anti BrdU (1∶200, Sigma). All the secondary antibodies were donkey antibodies purchased from Jackson ImmunoResearch and were used at 1∶200.

The slides were imaged using a Leica TCS SP5 confocal microscope system (20X glycerol lens N.A. 0.7; 63X glycerol lens N.A. 1.3) and the images were further processed in ImageJ and/or Photoshop. Images presented here were either single focal plane scans or a maximum-intensity projection of a stack of images. Some images were montages of multiple fields of view. The controls and the experimental samples were imaged and processed in exact the same way to ensure the final images were comparable.

### RNA Processing

The ONH including the initial segment of the myelination transition zone was isolated free of sclera and meningeal sheath (Fig. 1B insert), and then immediately preserved in ice-cold RNAlater (Ambion). Due to the extremely small amount of tissue, two ONHs had to be combined to yield enough RNA for downstream microarray and qRT-PCR. Thus the left eyes and the right eyes of two mice were combined respectively to form one pair of experiment and control samples. The total RNA of the ONH was extracted using the RNeasy Plus Micro Kit (Qiagen). The quality and quantity of the total RNA were evaluated using the Agilent 2100 Bioanalyzer and the RNA 6000 Pico Kit (Agilent). To ensure reliable downstream amplification, only the RNA samples that had a RNA integrity number (RIN) greater than 9.0 and total RNA amount of more than 30 ng were further processed for microarray or qRT-PCR. The majority of the RNA samples included in this study had a RIN greater than 9.5. RNA concentrations, yields, and RINs are given in the Table S1.

### Microarray

The RNA samples were amplified, biotinylated, hybridized and imaged at the Microarray Core at Dana-Farber Cancer Institute. The naïve samples and the samples from 1 day to 3 weeks after crush were processed together in one batch, while the samples from 3 months after crush were processed in a second batch under the same conditions. Microarray data analyses were performed independently for these two batches of samples. Biotin labeled cDNA was generated using the Ovation RNA Amplification System V2 (NuGEN) and the Encore Biotin Module (NuGen). The labeled cDNA was hybridized to the GeneChip Mouse Genome 430A 2.0 arrays (Affymetrix) in an Affymetrix hybridization oven 645. An Affymetrix GeneChip Fluidics Station 450 was used for array washing and staining. An Affymetrix GeneChip Scanner 3000 was used for array scanning and generating scan data. The GeneChip Mouse Genome 430A 2.0 array is a single array that contains 22,600 probe sets representing 14,000 well-characterized mouse genes. It is essentially one of the two arrays in the GeneChip Mouse Genome 430 2.0 array that was used in several related studies [7], [18], [20]. The advantage of the 430A 2.0 array is that it includes most of the better-characterized genes on the 430 2.0 array but requires only half the amount of cDNA for hybridization. The scan data (*.cel files) have been deposited in Gene Expression Omnibus (accession number GSE40857).

There were five biological replicates for each condition, and each biological replicate was composed of two ONHs. The probe level analysis of the microarray data was performed using DChip software. The intensity of all the arrays was normalized to the average overall intensity using the “Invariant Set Normalization” method [21]. The expression values were determined in DChip using the “PM/MM difference model” [21].

The comparison of the samples was made in DChip. Our criteria for differentially expressed genes were 1) greater than 2-fold difference (90% lower confidence bound) between the two group means; 2) greater than 100 in absolute difference between the two group means; 3) two-tailed unpaired t-test p-value <0.05 (when compare with naïve samples), or two-tailed paired t-test p-value <0.05 (when compared between the crush samples and the contralateral control samples). In criterion 1, the “90% lower confidence bound” of fold change was a more conservative estimate of the fold change and was used to avoid selection of genes with large SEs relative to their expression values. Criterion 2 was used to filter out genes that had very low expression levels in both conditions in a comparison. The p-value in criterion 3 was only considered as a filtering device rather than the actual significance value due to the “multiple comparisons” issue associated with large microarray data. False discovery rate (FDR) was calculated to estimate the significance.

Hierarchical clustering was performed using DChip. Due to the wide range of the expression levels, each gene was standardized across all the arrays to a mean of 0 and a standard deviation of 1 before clustering. Other high level analyses such as gene function enrichment analysis and pathway finding were performed using DChip, DAVID Bioinformatics Database [22], [23], and Ingenuity Pathway Analysis.

### qRT-PCR

qRT-PCR was performed to validate the results from the microarray. Independent RNA samples were collected as described above and they passed the same quality control as the RNA samples used for microarray. Ten ng of total RNA was reverse-transcribed and linearly amplified using the WT-Ovation RNA Amplification System (NuGen) according to manufacturer’s instructions. Amplified cDNA was diluted 1∶50 and 2 µl of the diluted cDNA was used as template for qPCR. qPCR was performed using an Applied Biosystems StepOnePlus Real-time PCR system and SYBR Green PCR Master Mix (Applied Biosystems) in 10 µl reaction volumes with 200 nM primer concentrations. Parameters for qPCR were: 10 min at 95°C, followed by 40 cycles of 15 sec at 95°C and 1 min at 60°C. Some of the primers used in qPCR were chosen from the validated primers in PrimerBank (http://pga.mgh.harvard.edu/primerbank/), others were designed in Primer3 program (http://frodo.wi.mit.edu/). All primers were designed to span at least one exon-exon junction. Gapdh was used as endogenous control in all the qPCR reactions, because its expression levels were very stable across all samples in our microarray data.

The WT-Ovation RNA Amplification System shares the same amplification methodology as the Ovation RNA Amplification System V2 used for the microarray samples. We had performed independent tests using series dilutions of brain RNA and RNA extracted from different numbers of ONHs as starting materials, and confirmed the fidelity of linear amplification of both abundant and low copy number genes over a wide range (at least four magnitudes). To validate the results of the microarray, 113 genes were tested in qRT-PCR using independent ONH RNA samples from all time points after optic nerve crush. Similar results were observed in more than 90% of the qRT-PCR reactions. This indicated high reliability and reproducibility of the microarray data.

### Single-cell RT-PCR

ONHs from naïve GFAPpr-tomato mice or mice 3 days after optic nerve crush were dissected as described above. The protocol for single-cell RT-PCR from optic nerve astrocytes was an adaptation of a method we used before for retinal neurons [24], [25]. The ONHs were incubated for 20 min in HBSS containing 0.5 mg/ml papain (Worthington). The tissue was centrifuged gently and resuspended in HBSS containing 10% horse serum. Then the tissue was triturated with heat-polished Pasteur pipettes to dissociate it into single cells. Collection of single cells from this dissociate was performed on a Zeiss Axiovert microscope equipped with micromanipulators. Single cells were aspirated with glass micropipettes pulled on a Narishige vertical electrode puller. The single cells were then expelled into sterile PBS with 0.5% BSA for washing. Then the cells were aspirated with a fresh micropipette and transferred into a thin-wall reaction tube containing a mix of 3 pmol of each primer and reaction buffer for reverse transcription and first round PCR. The primer mix contained primers for GFAP (as a control that the selected cell was an astrocyte), myelin basic protein (MBP, as a negative control to rule out contamination by oligodendrocytes), CD45 (as a negative control to rule out contamination by microglia), and tenascin C (TnC). Reverse transcription and first round PCR was done in a single vial reaction with the Access RT-PCR kit (Promega). Parameters for reverse transcription and first round PCR were as follows: The cells were lysed in the buffer/primer mix for 1 min at 65°C, 2U AMV-RT and 2U Tfl polymerase were added, then 45 min at 48°C were allowed for reverse transcription followed by 19 cycles of 1 min at 94°C, 1 min 60°C, and 2 min 68°C. Second round PCR was done separately for every target using 1/60 of the first round reaction, 7.5 pmol of each primer, and 0.25 U AmpliTaq Gold polymerase (Applied Biosystems). After a 32 cycles of amplification the PCR products were analyzed on 2% agarose gels.

### D2.hGFAPpr-EGFP Mice

The level of glaucomatous damage in the D2.hGFAPpr-EGFP mice was graded based on the immunohistochemistry staining of SMI32 in retina flat mounts, and toluidine blue staining of optic nerve sections.

For each eye, the ONH was isolated and kept individually in RNAlater at −20°C. The flat-mounted and fixed retina was blocked with 4% normal donkey serum and 0.1% Triton X 100 in PBS at 4°C overnight, incubated with mouse anti SMI32 antibody (1∶500, Covance) at 4°C for 1 week, donkey anti mouse antibody (1∶200, Jackson ImmunoResearch) at 4°C for 3 days, and mounted with Vectashield mounting medium. The distal optic nerve (2–3 mm from the globe) was fixed in 2.5% gluteraldehyde and 2% formaldehyde in 0.1 M cacodylate buffer with 0.08 M CaCl_2_ at 4°C overnight, then washed in 0.1 M cacodylate buffer. The optic nerve was post-fixed for 1.5 hours in 2% aqueous OsO_4_, dehydrated in graded ethanols, transitioned in propylene oxide, infiltrated with propylene oxide and epon mixtures (Tepon resin, Tousimis, USA), embedded in epon and cured for 24–48 hours at 60°C. One-micron sections were cut in a plane perpendicular to the axis of the nerve on a Leica Ultracut UCT and stained with 1% toluidine blue in 1% borate buffer. Images tiling the whole retina or the whole optic nerve were taken with an Olympus BX51 microscope with an Olympus DP70 camera.

The glaucomatous eyes exhibit sectorial loss of SMI32-IR in flat-mounted retinas [14]. Based on the amount of SMI32-IR loss in the retina, the retinas were graded to have no glaucoma, moderate glaucoma or severe glaucoma [26]. As an independent measure, the amount of axon loss in the optic nerve was also graded to these three levels. Three or four ONHs that had the same grade level in both the retina and the optic nerve were pooled together to form one sample. RNA extraction, quality control, reverse transcription, amplification and qRT-PCR were conducted as described in the above sections.

## Results

### The Glial Composition at the Mouse ONH

The mouse ONH differs from the human ONH in that it does not have a collagen-rich lamina cribrosa. Instead, the astrocytes form a honeycomb-like beamed structure that has been named glial lamina. Astrocytes are the main component of the glial lamina but it also contains microglia and the unmyelinated RGC axons (Figure 1B). The distribution of the astrocytes, the microglia, and the oligodendrocytes in a normal mouse ONH is shown in Figure 1C–F. Thus, even though astrocytes are by far the predominant cell type in the glial lamina, they are not the only type. Our microarray analysis therefore comprises a mixed population of cells. In other systems, it is possible to purify astrocytes by immunopanning [20], [27]. However, one needs relatively large amounts of starting material, such as can be obtained from dissociates of cerebral cortex with large amounts of protoplasmic astrocytes. The amount of starting material in the glial lamina is so limited, that we could not further purify the individual cell types. On the other hand, isolating the cells from the glial lamina has the advantage that the astrocytes in this preparation are already highly enriched and they are free of neuronal cell bodies. The optic nerve was crushed at about 1 mm behind the globe (Fig. 1 A and B), and the ONH including the initial segment of the myelination transition zone was isolated to study the molecular and cellular responses of the glial cells (Fig. 1 B and F).

### The Time Course of Retinal Ganglion Cell Degeneration

**Figure 2:**
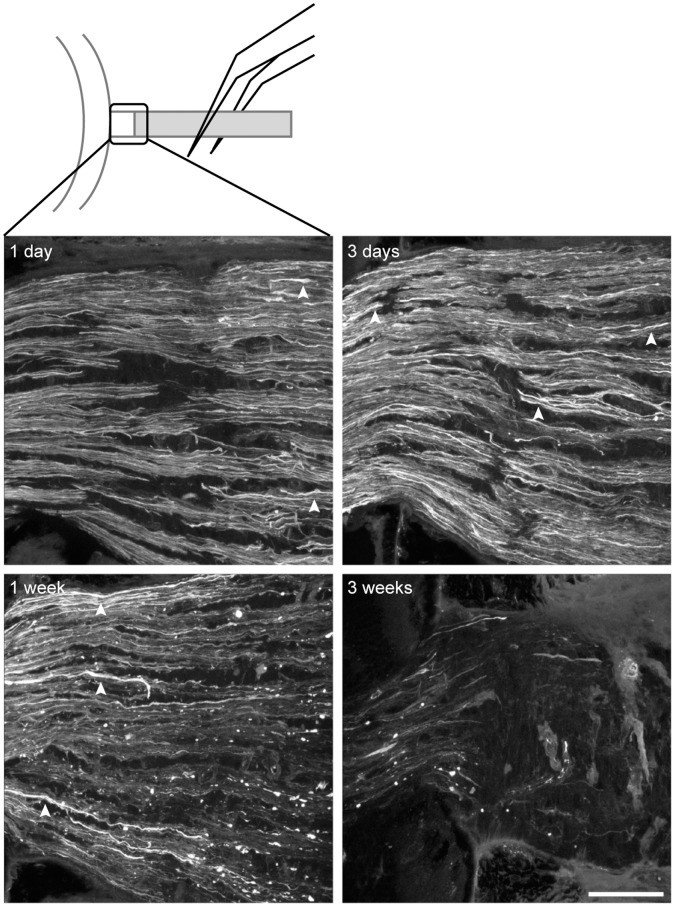
The timeline of retinal ganglion cell axon degeneration after optic nerve crush. The localization of the crush site and the glial lamina, where the microscopic images were taken, are indicated in the cartoon. Ganglion cell axon degeneration at the ONH was evaluated by immunohistochemistry against neurofilament SMI32. The retina is to the left of the image, and the crush site is to the right of the image. The axon swelling (arrowheads) was already visible at the distal end (right edge of the image) of the ONH 1 day after the crush, and spread to the proximal end (left edge of the image) at 3 days. The axon degeneration became severe at 1 week with most of remaining axons appearing swollen and fragmented. By 3 weeks, almost all the axons were lost. Scale bar, 50 microns.

The degeneration of the RGCs was investigated both at the axon level and at the soma level. Only the left optic nerve was crushed; the right served as control. There was also a group of naïve control mice that did not have any surgery done on either eye. Mice were sacrificed 1 day, 3 days, 1 week, 3 weeks and 3 months after the crush. The degeneration of the ganglion cell axons in the ONH region was evaluated by immunohistochemistry against non-phosphorylated neurofilament H (SMI32) in longitudinally sectioned optic nerves. As shown in Figure 2A, the swelling of the ganglion cell axons was already apparent at the distal end of the ONH 1 day after crush, but the proximal end appeared normal. The axon swelling spread to the whole ONH by 3 days and some fragmentation could be observed. Judging from the volume of the optic nerve, more than half of the axons were lost at 1 week, and the remaining half appeared swollen and fragmented. By 3 weeks, SMI32 immunoreactivity (SMI32-IR) had mostly disappeared from the ONH region, indicating almost total axon loss.

**Figure 3:**
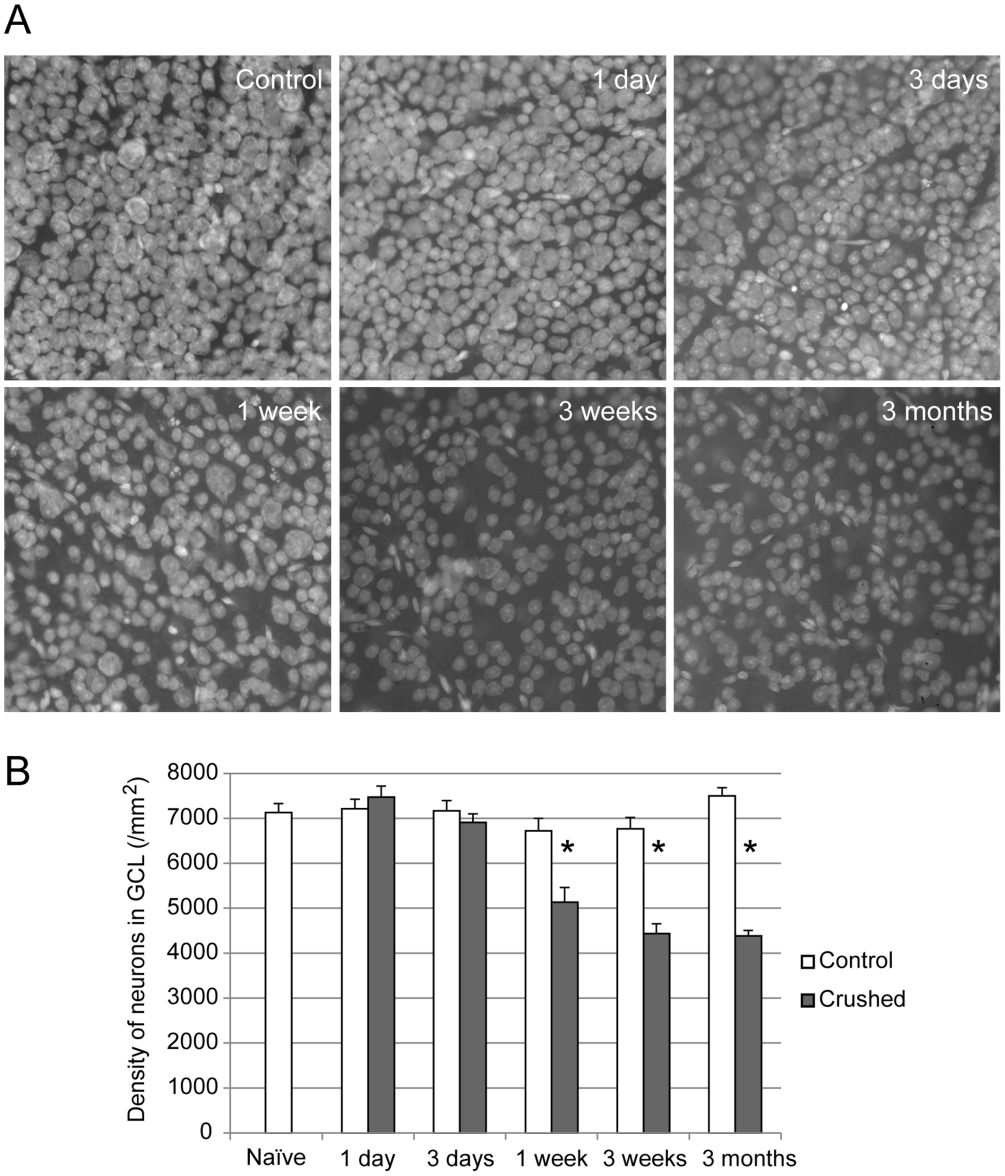
The loss of ganglion cells in the retina. A. Ganglion cell soma degeneration was estimated by counting the neurons in the mid-peripheral retina with the nuclear dye To-Pro1. B. The crushed eyes had a clear reduction 1 week after crush and reached a plateau (about 40% neuron loss in ganglion cell layer) by 3 weeks. The contralateral eyes were not different from the naïve controls at any time point. (* Two-tailed paired t-test p<0.05).

The RGC loss in the retina was estimated by counting the number of To-Pro-1 stained nuclei in the ganglion cell layer, which counts RGCs and displaced amacrine cells in the ganglion cell layer (Fig. 3A). We counted retinas from 10–14 mice for each condition. In the naïve mice, the density of the neurons in the ganglion cell layer in the mid-periphery retina was 7127±201 per square millimeter (Mean ± SE), similar to the previously published data [28]. In the operated eyes, significant drop out of the neurons began within 1 week after injury, reached 40% neuron loss by 3 weeks, and did not decrease thereafter (Fig. 3B). The density of the neurons in the contralateral control eyes did not change throughout the 3 months. Considering that the ganglion cell layer is composed of 40% retinal ganglion cells and 60% displaced amacrine cells [28], and amacrine cells are generally not affected by the optic nerve crush injury, the large majority of the RGCs were gone by 3 weeks.

### Gene Expression in the ONH Responded Strongly to the Crush Injury, but Largely Returned to Resting Levels with Time

The gene expression profile of the ONH tissue was analyzed using Affymetrix Mouse Genome 430A 2.0 arrays. Table 1 shows the number of differentially expressed gene probe sets out of the 22,600 gene probe sets included in the array. Strong gene expression changes were observed after optic nerve crush. A total of 1383 gene probe sets had greater than 2 fold changes (90% lower confidence bound, two-tailed paired t-test p<0.05) 1 day after crush, and the number decreased with time suggesting recovery. In contrast, only 10 probe sets had greater than 2 fold differences (90% lower confidence bound, two-tailed unpaired t-test p<0.05) between the naïve control eyes and the contralateral control eyes at any time points after injury. Thus, crushing the optic nerve in one eye did not significantly affect the contralateral eye.

**Table 1:** Number of differentially expressed gene probe sets (greater than 2 fold differences at 90% lower confidence bound, two-tailed t-test p<0.05) at different time points.

Time after crush		1 day	3 days	1 week	3 weeks	3 months
No. of probe sets different between naïve controls and contralateral controls		1	0	4	5	N/A
No. of probe sets different between crushed eyes andcontralateral controls		1383	845	788	240	127
False discovery rate (FDR)	Median	0.0%	0.0%	0.1%	0.0%	0.8%
	90^th^ percentile	3.4%	0.8%	2.7%	2.1%	5.5%

Optic nerve crush changed the expression levels of many genes in the crushed eyes, but did not affect the contralateral eyes.

We performed a second microarray study to investigate whether the genes were able to return to the resting level long after the injury. We found only 127 probe sets that remained differentially expressed between the crushed eyes and the contralateral control eyes 3 months after the crush. Analysis of these genes using the DAVID Bioinformatics Database identified no significantly enriched functional pathways. However, a manual read revealed that many of the down-regulated genes were involved in myelination (myelin basic protein, myelin-associated glycoprotein), axon cytoskeleton (neurofilaments, spectrins), axonal transport (kinesin family), and axon regeneration (growth associated protein 43). This loss of axon-structure genes was consistent with the total loss of axon in the optic nerve observed with SMI32-IR. Interestingly, some immune and phagocytic genes (macrophage scavenger receptor 2, T-cell receptor beta, complement component 3), growth factors (insulin-like growth factor 1, growth differentiation factor 5, bone morphogenetic protein 5) and adhesion molecules (cadherins) were up-regulated. This suggested that active repairing of the tissue was still underway at this point to clean up the debris and rebuild the tissue with glial cells.

### Validation of the Microarray Data by qPCR

To validate the microarray results, we tested 113 different genes that were found to be differentially regulated after optic nerve crush in our microarray hybridization data. For this validation experiment, independent optic nerve crushes were performed, mice were sacrificed at various time points same as in the microarray experiment, and RNA was extracted, reverse transcribed, and used in qPCR. All the genes that showed significant up or down regulation (satisfied all the three criterions for differential expression) in the microarray results had the same direction of change in the qPCR, indicating that the microarray expression data were highly reliable. It should be noted that it was common that these genes were only expressed differently at some but not all the time points after nerve crush. At the time points that these genes showed no changes in microarray (did not satisfy all three criterions for differential expression), the qPCR results also had minimal differences. Occasionally (7% of the cases) the qPCR results of the genes with less than 2 fold differences in the microarray projected to a different direction (e.g. a 20% reduction instead of a 20% increase), but since the changes were less than two folds and thus were considered as “no change” in either case, the microarray results were still valid. This further supported our stringent criterions for defining significant changes in microarray analyses, which ensured the high reliability of our interpretation of the data. Based on the high reproducibility of the microarray data and to avoid redundancy, the microarray data were plotted in most of the figures here except where noted otherwise.

### The Time Course of Changes in Glial Molecular Markers

**Figure 4:**
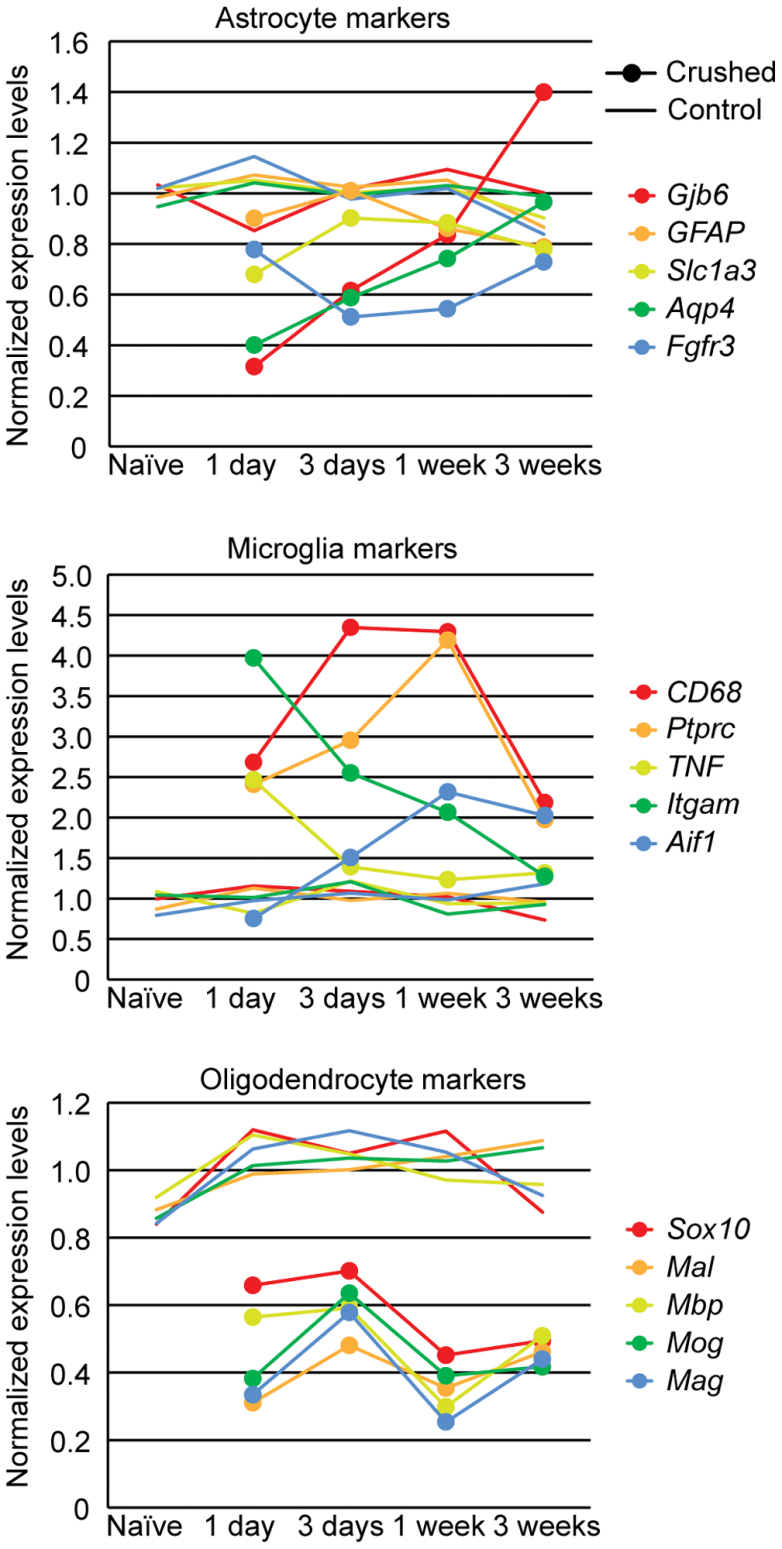
Microarray data of the expression levels of glial marker genes after crush. Overall, there was significant down-regulation in astrocyte markers and up-regulation in microglia markers, but both returned to baseline levels at 3 weeks. The down-regulation in oligodendrocyte markers did not recover. The smooth lines represent the naïve and contralateral control samples and the lines with dot markers represent crushed samples. They were both normalized to the mean of all the naïve controls and the contralateral controls of the same gene.

As shown in Figure 1 C–F, the major glial cell types at the ONH and the myelination transition zone were astrocytes, microglia, and oligodendrocytes. They each responded uniquely to the crush injury. The cell marker genes for astrocytes, microglia and oligodendrocytes were selected according to a recent publication [20]. For each gene, the mean of all the naïve controls and the contralateral controls was taken as the baseline expression level. The mean of the five biological replicates for each condition was normalized to this baseline level. The naïve controls and the contralateral controls did not deviate from the baseline for more than 20% at any time points in all three cell types. In contrast, the crushed samples showed great cell type specific responses (Fig. 4).

Overall, the astrocyte markers were down-regulated after the crush (Fig. 4, top panel). Gap junction membrane channel protein beta 6 (*Gjb6*) and aquaporin 4 (*Aqp4*) had 60–70% reductions 1 day after crush, and gradually returned to the baseline levels by 3 weeks. This agreed with our previous observation on the astrocytes’ morphological remodeling after nerve crush – the astrocytes shed membrane channels and lose intercellular connections before they become mobile, and regain the membrane connections as they recover. Interestingly, *Gfap* did not increase its expression level as the astrocytes became reactive at the ONH. *Gfap* is commonly regarded as a reactive astrocyte marker. In the brain, it is expressed at very low level under normal conditions, but increases significantly during astrogliosis. In contrast, *Gfap* is already expressed at a high level in the normal ONH, and it did not further increase in our crush model and in two glaucoma models [17], [18], [29]. All the microglia markers were up-regulated after crush (Fig. 4, middle panel). Tumor necrosis factor (*Tnf*) and integrin alpha M (*Itgam*) peaked the first day after crush, while allograft inflammatory factor 1 (*Aif1*, also known as *Iba1*) and CD68 antigen (also known as *ED1*) did not reach the peak until later. By 3 weeks, they all fell back to close to resting levels. The oligodendrocyte markers such as myelin basic protein (*Mbp*) and myelin oligodendrocyte glycoprotein (*Mog*) reduced their expression levels in synchrony (Fig. 4, bottom panel). They remained down-regulated for 3 weeks and did not return to the resting levels as the astrocyte markers and microglia markers did. At 3 months, all the astrocyte markers and microglia markers were at baseline levels, but the oligodendrocyte markers were still down-regulated (data not shown).

### The Time Course of Glial Morphological Remodeling

**Figure 5:**
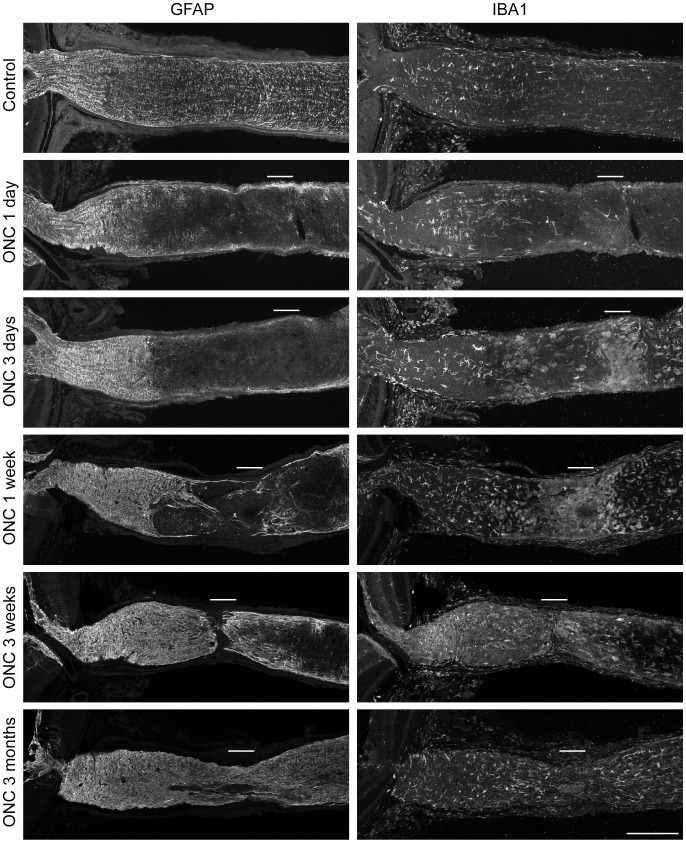
The morphology of astrocytes and microglia after crush. The localization of the crush sites is indicated with white bars. Astrocyte at the crush site degenerated and formed a GFAP-IR free zone within a few days. Astrocytes from both the proximal and distal sides of the optic nerve then migrated into this zone and rebuilt the architecture. The GFAP-IR at the ONH intensified after crush, indicating reactive astrogliosis. There were high density of IBA1-IR positive microglia cells/macrophages at the crush site. They were strongly activated and had retracted processes and enlarged cell bodies. This reactive morphology resolved with time. The microglia located further away from the crush site, both distally and proximally, including the ONH, retained stratified morphology. Scale bar, 200 microns.

The optic nerve was stained with antibodies against astrocyte, microglia and oligodendrocyte markers (GFAP, IBA1 and MBP, respectively) (Fig. 5). GFAP-IR showed clear reduction at the crush site 1 day after the crush, indicating degeneration of astrocytes due to direct mechanical insult. A GFAP-free zone had formed as a result of degeneration three days after crush. This zone was about 0.5–0.8 mm in length and it spread to both the proximal and distal sides of the crush site, similar to what was observed in earlier studies [30]. By 1 week, astrocytes began to migrate back into the GFAP-free zone from both ends. The gap was partially filled by 3 weeks, and completely filled by 3 months, indicating that a mature glial scar had formed. At the ONH, 1 mm proximal to the crush site, the GFAP-IR intensified after the crush, indicating astrocyte hypertrophy. Since the mRNA level of *Gfap* did not change at the ONH, this increase in protein level was most likely mediated by post-transcriptional mechanisms.

IBA1-positive cells, which can represent either microglia or macrophages, responded very differently from the astrocytes (Fig. 5). There were a great number of IBA1-IR positive microglia/macrophages at the crush site 3 days to 1 week after injury. They had typical reactive morphology, including retracted processes and enlarged cell bodies. Microglia/macrophages that were located further away from the crush site, such as at the ONH and at the distal end of the optic nerve, did not show hypertrophy or reactive morphology. Their processes remained elongated and stratified. However, since the microglia/macrophages at the ONH had strong reactive molecular profile (Fig. 4), they were still likely undergoing gliosis. The reactive morphology at the crush site gradually resolved and the microglial/macrophage cells returned to close to the normal appearance by 3 months.

Based on the MBP-IR, the degeneration of oligodendrocytes began at the crush site just 1 day after crush and it spread both distally and proximally as time went by. It never returned to the resting level. (Data not shown).

### Genes and Pathways Responded to the Injury with Different Time-dependent Patterns

**Figure 6:**
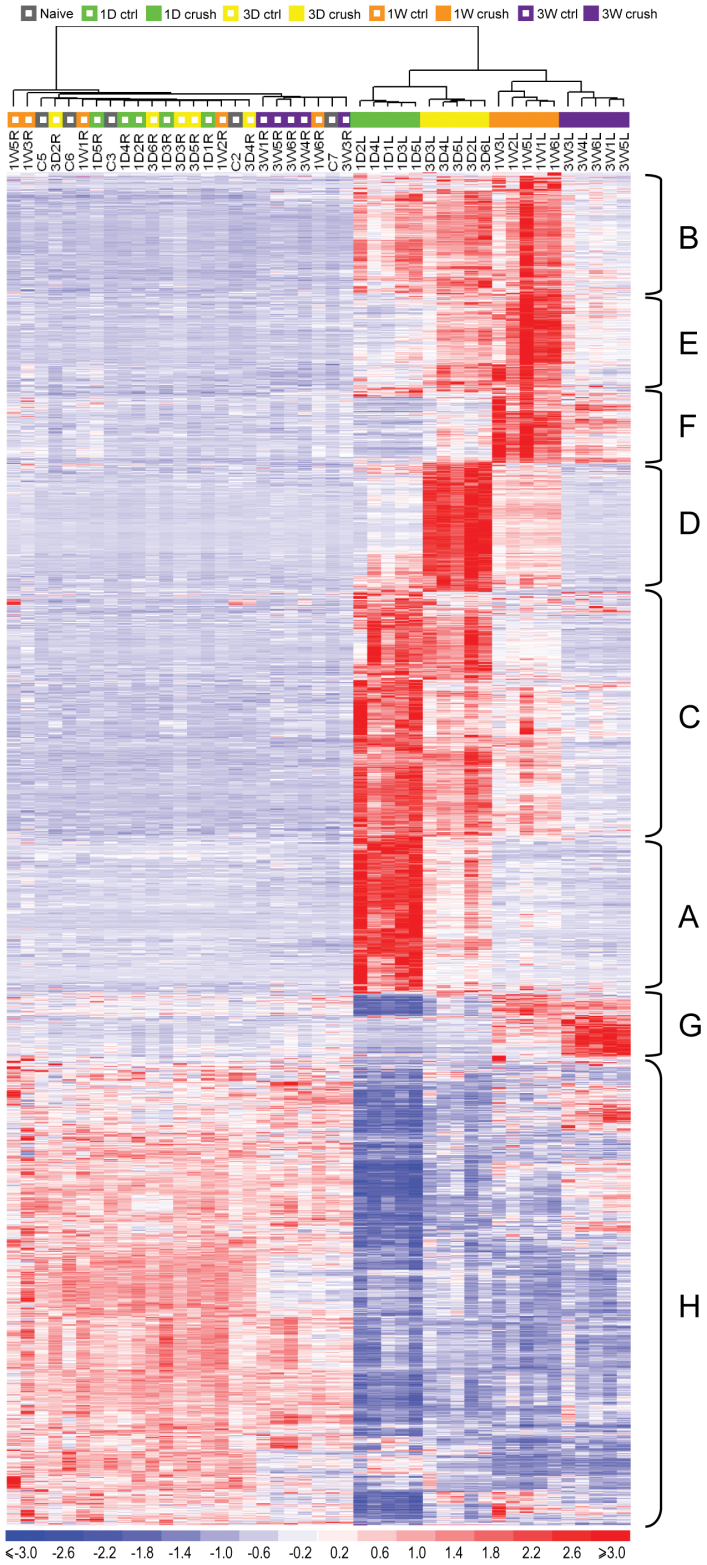
Clustering analysis of the samples and the genes based on the 2056 probe sets that were differentially expressed after crush. Each column is one array sample. Each row is one probe set that had been normalized to have mean 0 and standard deviation 1. The crushed samples (filled squares above the heatmap) from each time point formed unique subgroups before merging into one big cluster, which was distinctly different from the cluster of the naïve and the contralateral controls (open squares). The genes changed expression levels in a time dependent manner. They were divided into 8 major groups (A–H) based on their expression patterns. The pathways that were enriched in each group were listed in Table 2.

Many genes were differentially expressed at multiple time points. When the data from 1 day to 3 weeks were consolidated, there were a total of 2056 unique probe sets that had greater than 2-fold differences at any time point from 1 day to 3 weeks. These probe sets were used to cluster the samples and to generate the heatmap shown in Figure 6. The high similarity among the 5 biological replicates under each condition assured the reliability of the data. All the 5 naïve control samples and the 20 contralateral control samples shared similar profiles and fell in one cluster group, indicating that there was no difference among the controls at different time points. In contrast, the crushed samples at each time point clustered as distinct subgroups before combining into one big group that was clearly divided from the control group.

**Figure 7:**
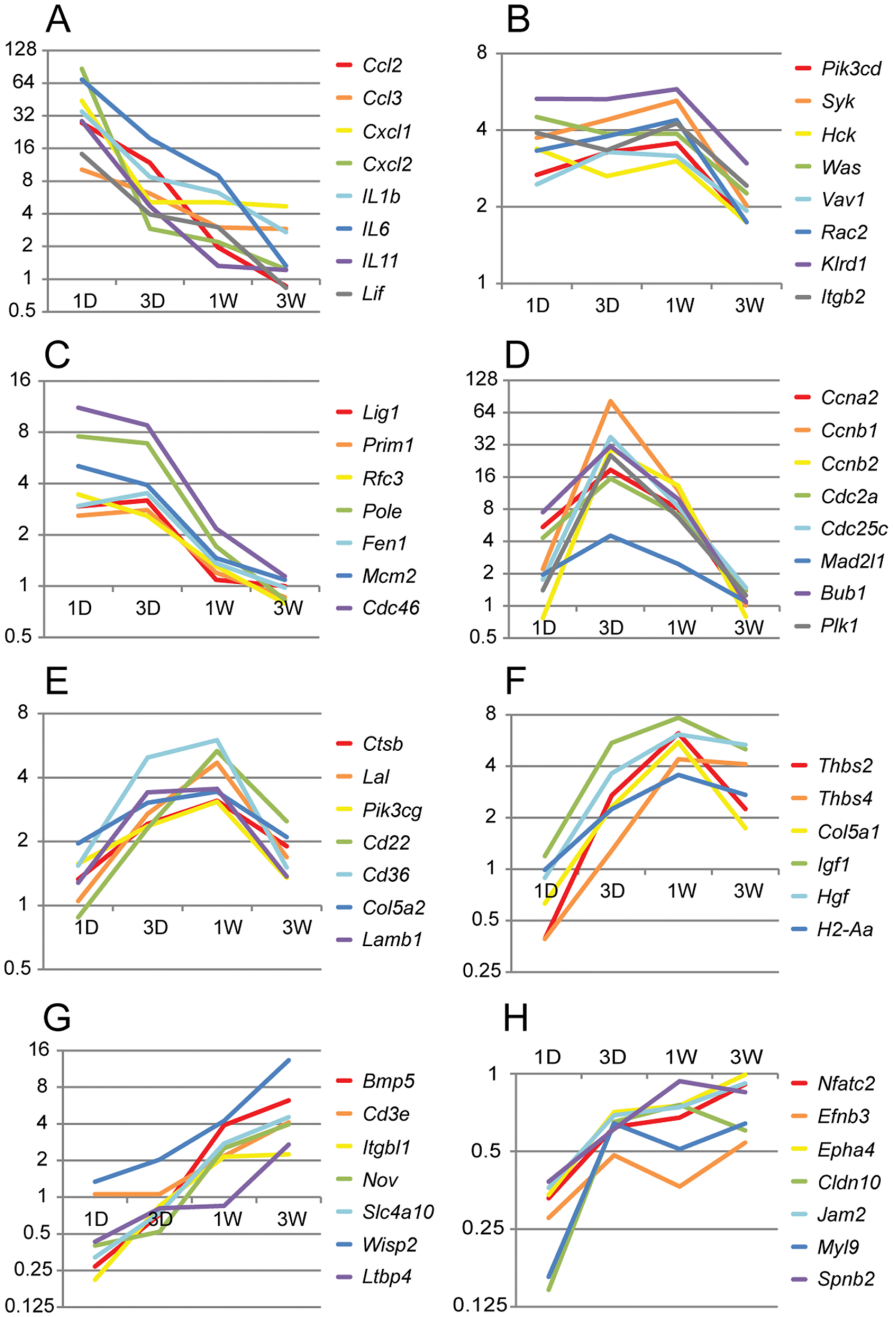
Microarray data of the expression levels of selected genes in the enriched pathways. Panels A–H corresponded to Groups A–H in Figure 5 and Table 2. The x-axes were time points after crush −1 day (1D), 3 days (3D), 1 week (1W), 3 weeks (3W), and the y-axes were mean fold changes between the crushed samples and the contralateral controls.

These genes clearly had different time-dependent expression patterns. Some genes were up-regulated immediately after crush and remained up-regulated for at least 1 week, while other genes did not respond until 1 week after the crush. Based on the temporal changes in expression pattern, these genes were clustered and divided into 8 major groups (Groups A–H in Fig. 6). To find out whether genes in the same group share similar biological functions, Functional Annotation Analysis was performed on the genes from each group using the DAVID Bioinformatics Database. The significantly enriched KEGG pathways and their p-values were listed in Table 2. The fold changes of selected genes in these pathways were shown in Figure 7.

**Table 2:** List of pathways that responded in different patterns after crush.

Group	KEGG Pathway	P-Value
**A**	Cytokine-cytokine receptor interaction	1.20E-11
	NOD-like receptor signaling pathway	6.70E-07
	Chemokine signaling pathway	1.40E-05
	Cytosolic DNA-sensing pathway	2.90E-04
	Toll-like receptor signaling pathway	8.00E-03
	Hematopoietic cell lineage	2.10E-02
	Jak-STAT signaling pathway	3.80E-02
**B**	Natural killer cell mediated cytotoxicity	5.40E-06
	Fc gamma R-mediated phagocytosis	6.60E-06
	B cell receptor signaling pathway	8.50E-05
	Fc epsilon RI signaling pathway	1.00E-03
	Leukocyte transendothelial migration	1.40E-03
	Chemokine signaling pathway	4.00E-03
	Toll-like receptor signaling pathway	1.30E-02
	Primary immunodeficiency	1.60E-02
	Apoptosis	3.10E-02
**C**	DNA replication	6.80E-14
	Cell cycle	1.90E-05
	Mismatch repair	3.30E-03
	Nucleotide excision repair	6.10E-03
	Base excision repair	9.00E-03
	Pyrimidine metabolism	1.30E-02
	Chemokine signaling pathway	1.90E-02
	Purine metabolism	3.70E-02
	Cytokine-cytokine receptor interaction	5.00E-02
**D**	Cell cycle	2.60E-10
	Oocyte meiosis	1.40E-08
	Progesterone-mediated oocyte maturation	3.50E-07
	p53 signaling pathway	3.10E-05
**E**	Lysosome	7.10E-04
	Hematopoietic cell lineage	9.50E-03
	B cell receptor signaling pathway	3.40E-02
	ECM-receptor interaction	3.90E-02
**F**	Focal adhesion	2.00E-04
	ECM-receptor interaction	4.30E-04
	Antigen processing and presentation	4.30E-02
**G**	None	
**H**	Drug metabolism	2.80E-03
	Steroid biosynthesis	6.40E-03
	Axon guidance	1.30E-02
	Tight junction	3.90E-02

Functional annotation analysis was performed using genes in each of the eight groups (A–H) in Figure 5. The most enriched KEGG pathways and their enrichment p-values are listed here. Many of these pathways were involved in the inflammation and immune response, and cell proliferation.

Group A and Group B were dominated by the immune and inflammation related pathways. The differences were that genes in Group A were mostly involved in the innate immune system and showed more acute responses. They strongly increased in expression levels immediately after the crush, but the magnitudes dropped sharply within days. In contrast, Group B contained genes related to both the inflammation and the adaptive immune system. The up-regulation was not as strong as it was in Group A, but the elevated expression levels were sustained for at least 1 week before returning toward baseline levels. Group C mostly represented DNA replication and repair pathways, while Group D was mostly involved in cell cycles. Compared to Group C, genes in Group D had more delayed onset, but stronger amplitudes. Groups E and F were turned on even later in the response process. Genes in these two groups appeared to be involved in debris cleaning and tissue reconstruction. Groups G and H were the only two down-regulated groups. Genes in Group G were not enriched in any particular pathways, but they had interesting patterns with an inverse from a down-regulation at 1 day to an up-regulation at 3 weeks. Genes in Group H were enriched in metabolism and biosynthesis. They had a sharp drop in expression levels immediately after crush, and then either remained at low levels or returned close to the resting levels.

In summary, genes involved in the innate immune system and the DNA repair mechanisms were among the first responders to the crush injury. They were soon followed by inflammation, adaptive immune system, and cell proliferation. Debris removal and tissue reconstruction began within 1 week after crush and could last for more than 3 weeks.

In the above approach, genes in the same pathway but responding with different time courses were split into different groups, e.g. cell cycle and chemokine signaling pathway were enriched in multiple groups. We performed additional Functional Annotation Analysis using all the 2056 probe sets that were differentially expressed at any time point after the injury. Cell proliferation, extracellular matrix-receptor interaction, and the inflammation and immune responses were the most enriched KEGG pathways (Table 3). Similar results were obtained with GenMAPP pathways (Table 4).

**Table 3:** List of the most enriched KEGG pathways and their enrichment p-values.

KEGG pathways	P-Value
Cytokine-cytokine receptor interaction	3.40E-09
Chemokine signaling pathway	9.00E-06
ECM-receptor interaction	1.50E-05
DNA replication	1.00E-04
Cell cycle	1.30E-04
Primary immunodeficiency	6.00E-04
Hematopoietic cell lineage	7.90E-04
Toll-like receptor signaling pathway	1.60E-03
p53 signaling pathway	2.20E-03
NOD-like receptor signaling pathway	2.80E-03

The analysis was based on all the 2056 probe sets that were differentially expressed from 1 day to 3 weeks after crush.

**Table 4:** List of the most enriched GenMAPP pathways and their enrichment p-values.

GenMAPP pathways	P-value
Matrix metalloproteinases	4.6E-05
DNA replication reactome	5.9E-05
Inflammatory response pathway	1.3E-04
Cell cycle	1.6E-04
TGF beta signaling pathway	2.4E-04
G1 to S cell cycle reactome	4.0E-04
Calcium regulation in cardiac cells	5.8E-04
Cholesterol biosynthesis	6.1E-04

The analysis was based on all the 2056 probe sets that were differentially expressed from 1 day to 3 weeks after crush.

### The Proliferating Cells were Microglia/macrophages

**Figure 8:**
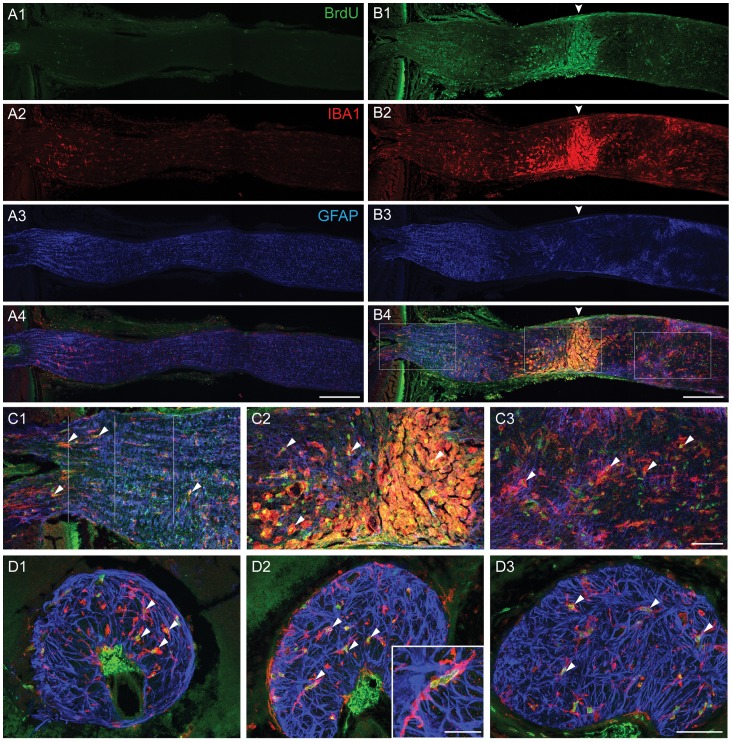
Microglia/macrophages proliferated after crush injury. Immunohistochemistry against BrdU, IBA1 and GFAP were performed in longitudinally sectioned optic nerves. A1–4. Contralateral control, no BrdU-IR was detected. B1–4. 1 week after crush, BrdU-IR colocalized with IBA1-IR. Arrowheads point at the crush site. C1–3. Magnified views of the three outlined regions in B4. C1 is the ONH, C2 is the crush site, and C3 is distal optic nerve. D1–3. Transvers sections of the ONH at the three locations indicated in C1. D1 is pre-lamina, D2 is the glial lamina, and D3 is post-lamina. Arrowheads in C1–3 and D1–3 point at colocalized BrdU-IR and IBA1-IR. The insert in D2 shows one microglia/macrophage with elongated BrdU-IR positive nuclei, indicating active cell division. Scale bars are 200 microns in A and B, 50 microns in C and D, and 20 microns in D2 insert.

As described above, DNA replication genes and cell cycle genes were among the most up-regulated genes after optic nerve crush. To find out the identity of the proliferating cells, daily intraperitoneal injections of BrdU were given to the mice starting immediately after crush and lasting for up to 1 week. This duration was chosen because the up-regulation of the genes only lasted for up to 1 week. The mice were sacrificed at various time points and the optic nerve was stained with antibody against BrdU (Fig. 8). BrdU-IR was not detectable in the contralateral control optic nerve at any time point, indicating absence of proliferation under normal conditions. BrdU-IR was already present in many cells in the crushed nerve 2 days after injury, and a lot more BrdU-IR positive cells were detected at later time points. This confirmed that the proliferation indeed started soon after the injury and lasted for at least 1 week. These proliferating cells were distributed throughout the length of the optic nerve both proximal and distal to the crush site, but the highest density was observed right at the crush site.

Most of the BrdU-IR localized to the nuclei of the IBA1-IR positive cells. Some of these nuclei appeared to be elongated suggesting active cell division. Rarely there were BrdU-labeled nuclei that did not colocalize with IBA1-IR. Based on the MBP-IR, oligodendrocytes did not regenerate at any time points. Astrocytes indeed started repopulating the GFAP-free zone at around 1 week, and some BrdU-IR appeared to colocalize with GFAP-IR. But due to the overlapping of the GFAP-IR from neighboring astrocytes, it was not possible to identify individual astrocytes and to locate their nuclei. Thus it remained inconclusive whether the repopulation was mediated by astrocyte proliferation, by migration of astrocytes from other regions, or both. It should be noted that the vast majority of the BrdU labeled cells were clearly identified as microglia/macrophages, the unidentified BrdU-IR positive cells were very rare events (Fig. 8).

### Genes Involved in Tissue Remodeling were Up-regulated

**Figure 9:**
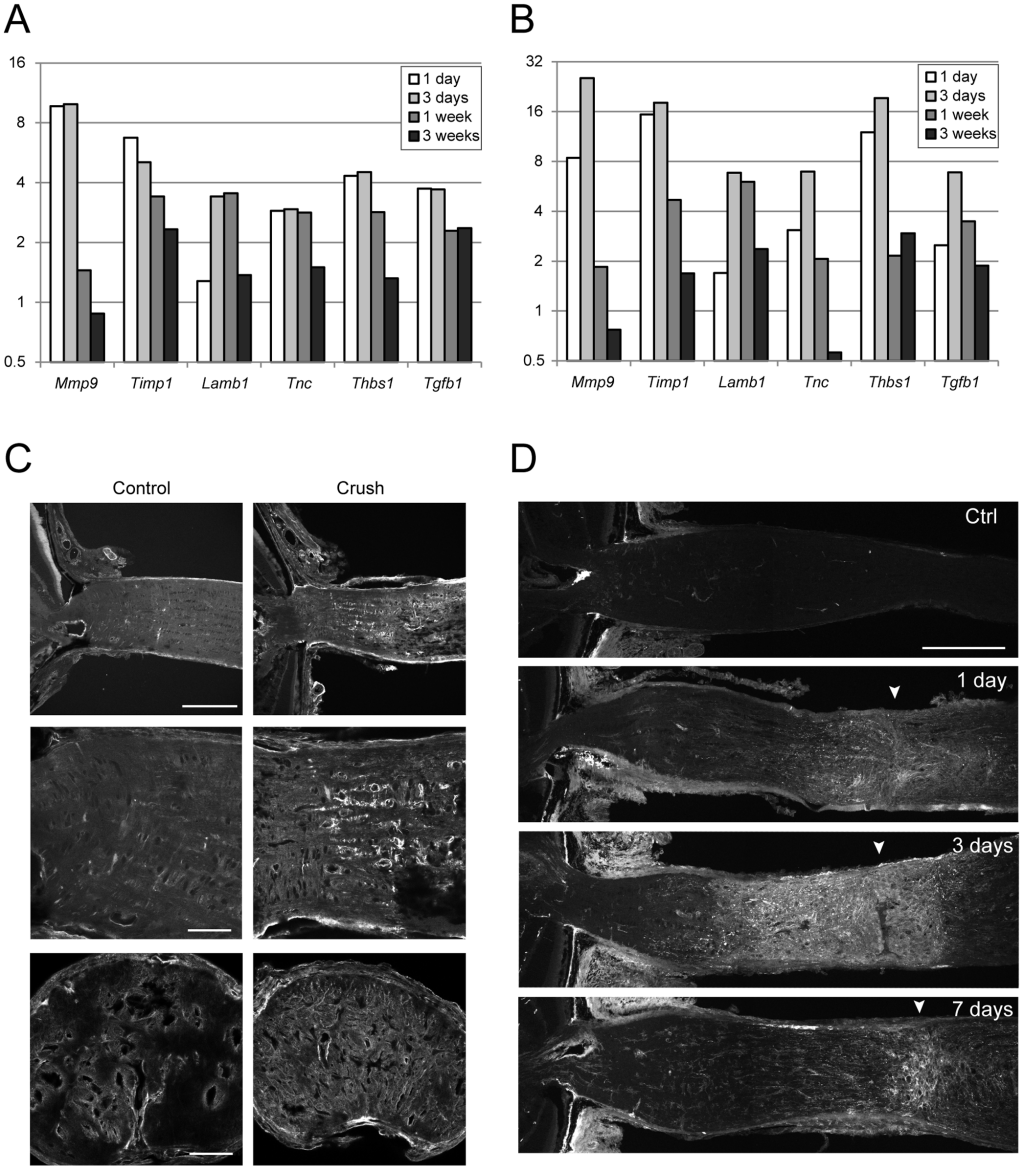
The up-regulation of tissue remodeling genes. A. Microarray data of the mRNA levels in crushed eyes normalized to the contralateral control eyes. B. qRT-PCR confirmation of the results in A. C. TNC protein levels increased in ONH at 3 days after crush. Images show the longitudinal sections at low magnification (first row, scale bar 200 microns) and at high magnification (second row, scale bar 50 microns), and the transvers sections through the unmyelinated glial lamina (bottom row, scale bar 50 mcirons). D. THBS1 protein levels peaked at 3 days after crush (scale bar 200 microns).

We previously reported that mouse ONH astrocytes go through a two-stage morphological remodeling after optic nerve crush [6]. This changes the architecture of the ONH. The extracellular matrix-receptor interaction pathway was among the most regulated pathways after crush (Table 3 and 4), supporting our previous findings. Quantitative RT-PCR confirmed the up-regulation of many genes involved in tissue remodeling, such as matrix metallopeptidase 9 (*Mmp9*), tissue inhibitor of metalloproteinase 1 (*Timp1*), laminin beta 1 (*Lamb1*), tenascin c (*Tnc*) and thrombospondin 1 (*Thbs1*) (Fig. 9).

**Figure 10:**
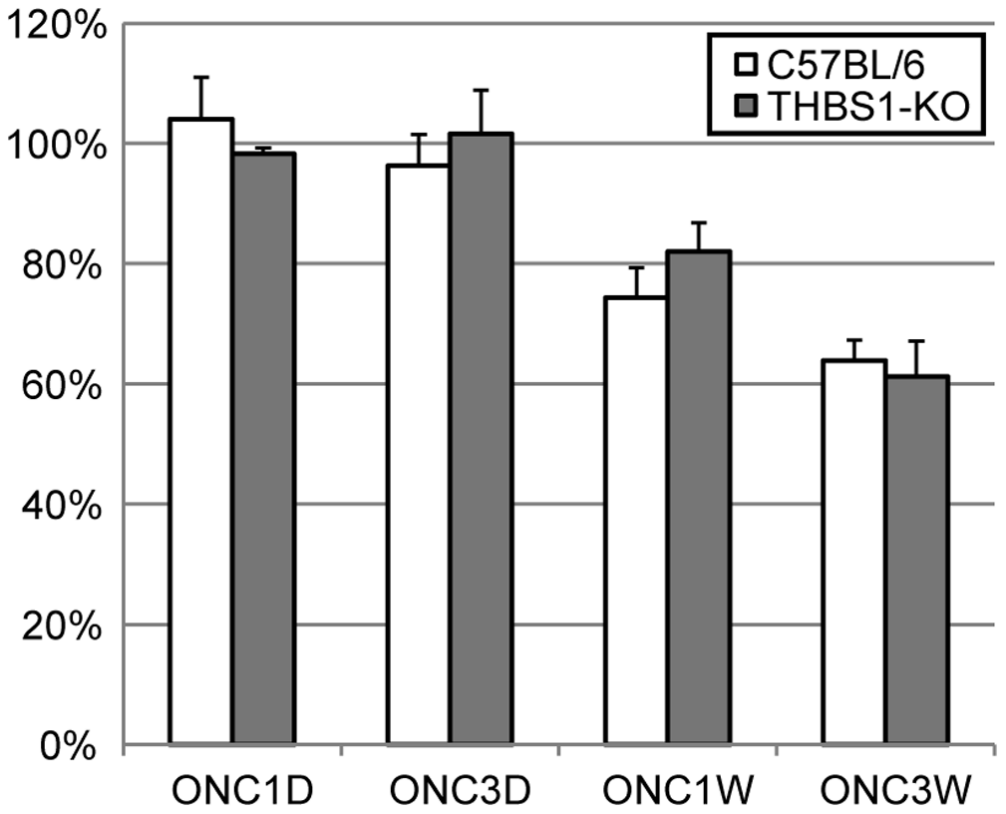
Ganglion cell survival after optic nerve crush in thrombospondin-1 knock out mice. Cells in the ganglion cell layer were counted in mid-periphery with the nuclear dye To-Pro1. Differences in cell survival between C57bl/6 and the thrombospondin-1 knock-out mice were not significant.

We also performed immunohistochemistry against TNC and THBS1 and confirmed the increase in their protein levels after optic nerve crush (Fig. 9 C&D). TNC can interact with extracellular matrix protein membrane receptors such as integrin, which regulates cell adhesion, migration, and proliferation. THBS1 is a matricellular protein that plays important roles in tissue genesis and repair. It interacts with cytokines, growth factors, matrix components, membrane receptors, and extracellular proteases, thus regulates inflammation, proliferation, and migration. THBS1 is a potent activator for anti-inflammatory transforming growth factor beta 1 (TGFB1), which was also up-regulated during the same time frame (Fig. 8). We hypothesized that THBS1 functioned through TGFB1 to keep inflammation in check and thus maintain homeostasis. However, to our surprise, the ganglion cell survival rate in THBS1 knockout mice was not significantly different from the C57BL/6 mice (Figure 10).

### The Heterogeneity of the Glial Population at the ONH and the Investigation of Cell Specific Responses

**Figure 11:**
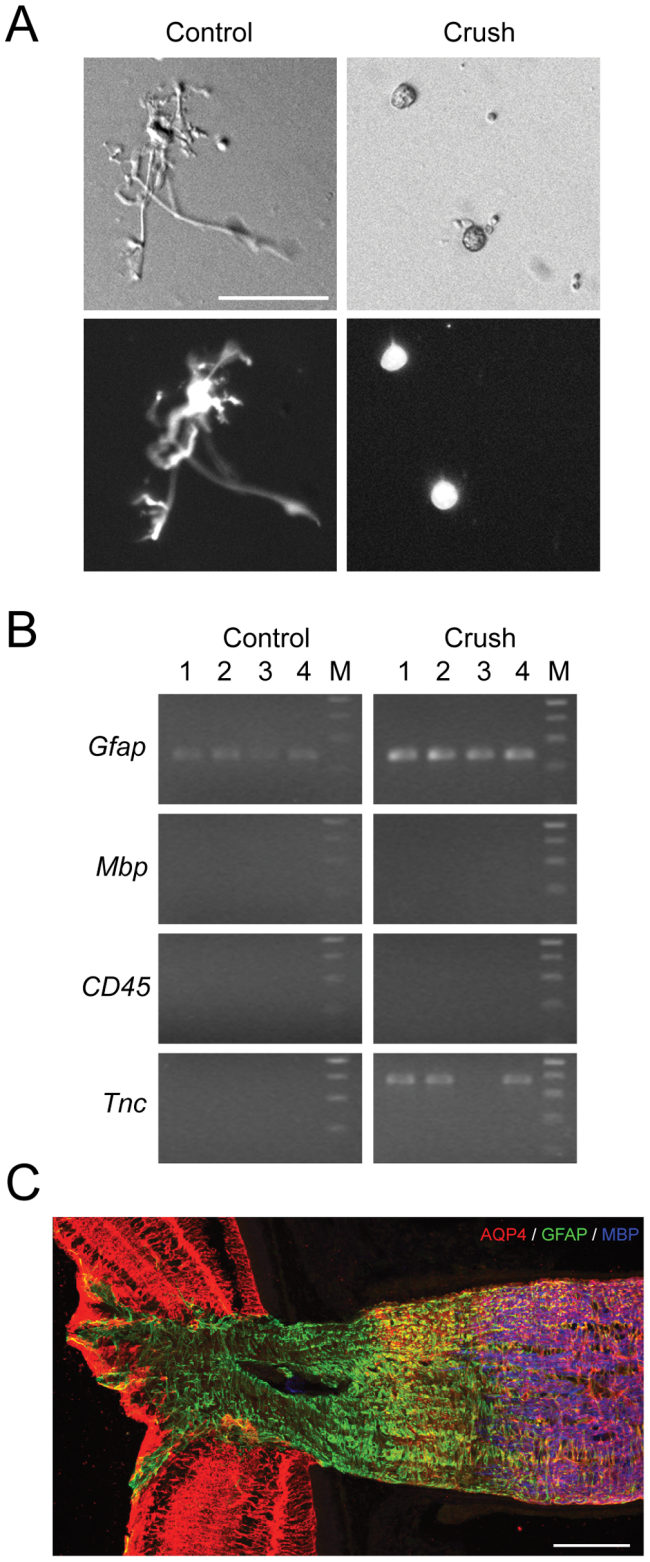
The heterogeneity of the glia cells at the ONH and the investigation of cell specific responses. A. Acutely dissociated ONH astrocytes from the crushed and the contralateral control eyes were collected for single-cell PCR. Astrocytes were identified based on the morphology and the expression of tomato marker (bottom row). B. Agarose gel showing results of single-cell RT-PCR. Presence of astrocyte marker (*Gfap*) and absence of microglia marker (*CD45*) and oligodendrocyte marker (*Mbp*) confirmed the identity of the cells as astrocytes. *Tnc* was not detectable in control astrocytes, but was highly expressed in most of the astrocytes after crush. C. Astrocyte marker AQP4 was expressed in the myelinated optic nerve, in the distal region of the ONH and in the retina, but not in the astrocytes in the proximal section of the ONH. Scale bar, 100 microns.

Our microarray data reflected molecular responses of a mixed population of glial cells against crush injury. To further understand the functions of each cell type, gene profiles of purer populations of each cell type would be necessary. One way to achieve this is to perform single-cell RT-PCR using acutely dissociated ONH glial cells. We performed a proof-of-principle study using GFAPpr-tomato mice to investigate the expression of *Tnc* in selectively collect ONH astrocytes. Under normal conditions, dissociated astrocytes could be easily distinguished from the other ONH cells based on their morphology. After optic nerve crush, however, the astrocytes often retracted their processes and became unidentifiable *in vitro*. In the GFAPpr-tomato mice, astrocytes express a red fluorescent protein that aids the identification of astrocytes after dissociation (Fig. 11A). *Gfap*, but not *Mbp* or *CD45*, was detected in all the collected single cells, further confirming the identity of the cells as astrocytes. *Tnc* was undetectable in astrocytes isolated from control ONHs, but became highly expressed after crush (Fig. 11B). This indicated that it was possible to use single-cell RT-PCR to investigate gene expression changes in specific cell types.

As we have described, our samples of the ONH and myelination transition zone included astrocytes, microglia and oligodendrocytes. It is possible, however, that the heterogeneity was actually greater than that. For example, AQP4 is a commonly used astrocyte marker, but a closer look at the AQP4-IR at the ONH revealed that not all the astrocytes at the ONH expressed AQP4 (Fig. 11C). While the astrocytes located in the myelinated optic nerve and in the distal half of the unmyelinated glial lamina strongly expressed AQP4, the astrocytes located in the proximal half (closest to the retina) of the unmyelinated glial lamina did not express AQP4 at all. Interestingly, the AQP4-IR free zone was only restricted to the proximal glial lamina, the astrocytes and the Müller glial cells in the retina both expressed AQP4. What caused this phenomenon was unclear. It was also unclear whether this indicated that the astrocytes at the proximal ONH were of a unique subtype. The function of AQP4 as a water channel and its intriguing expression pattern at the ONH may make it relevant in glaucoma.

### Reactive Gliosis in Different Injuries– Optic Nerve Crush vs. Glaucoma

DBA/2J mice spontaneously develop elevated intraocular pressure and retinal ganglion cell loss as they age, which closely mimics glaucoma. The age of onset and the severity of the phenotype vary greatly and some eyes may remain normal throughout the lifetime. We collected ONHs from 16–20 months old D2.hGFAPpr-EGFP mice to compare the molecular profiles of the reactive gliosis in glaucoma to the crush model. The eyes were divided to three categories – no glaucoma, moderate glaucoma and severe glaucoma – based on the SMI32-IR in the retina and the axon morphology in the optic nerve. Eyes in the no glaucoma category had normal SMI32-IR and healthy optic nerve that were not different from wild type mice. Eyes that had moderate glaucoma exhibited sectorial loss of SMI32-IR in the retina and 10–40% axon loss in the optic nerve. Eyes with severe glaucoma had lost more than half of the SMI32-IR and the axons.

**Figure 12:**
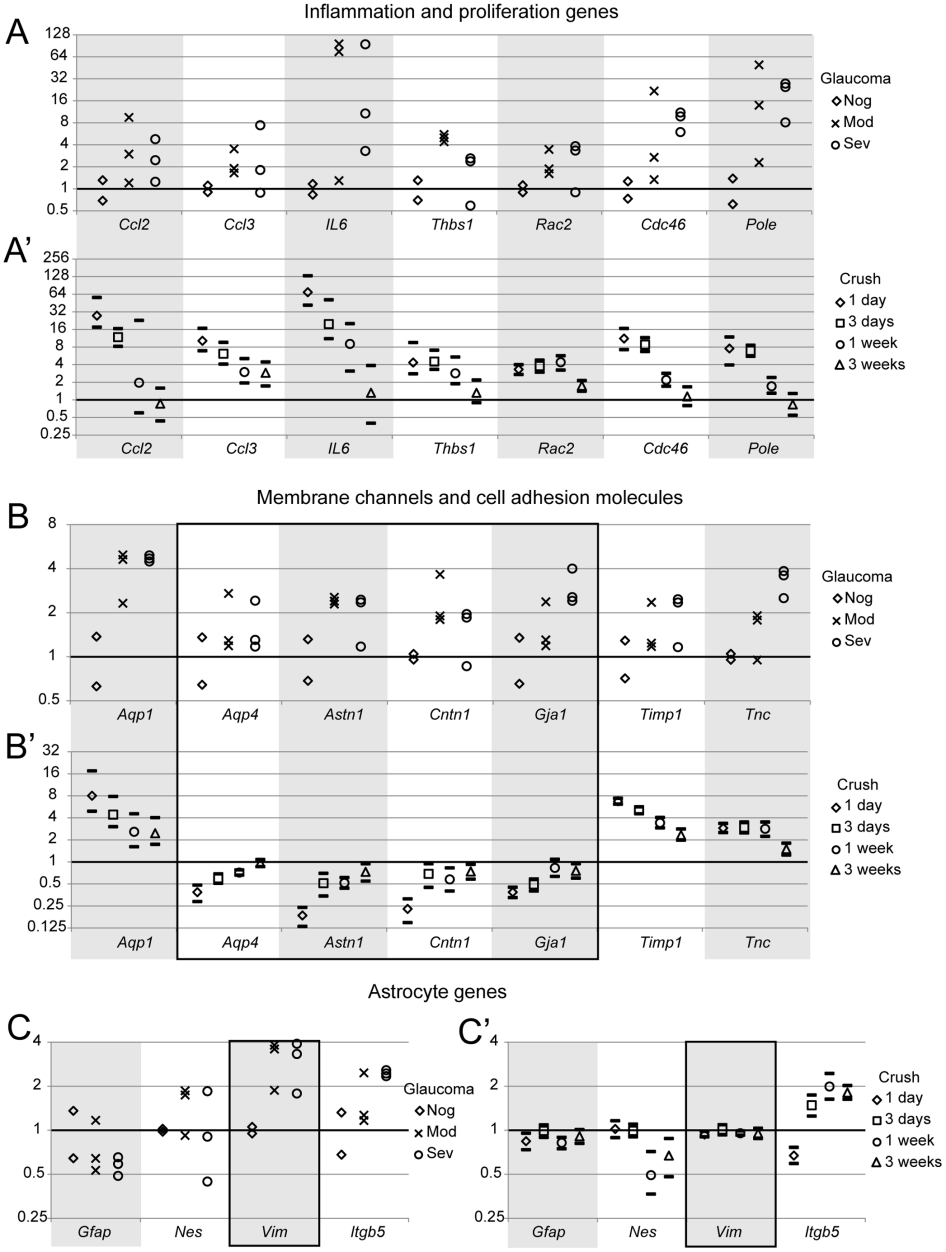
Comparison of gene expression levels between ONHs in D2.hGFAPpr-EGFP mice (A, B and C) and ONHs after crush (A’, B’ and C’). Gene expression levels in the D2 mice were determined by qRT-PCR. Each independent sample contained 3–4 ONHs with the same retina and optic nerve grades. There were total 2 no glaucoma (Nog), 3 moderate glaucoma (Mod) and 3 severe glaucoma (Sev) samples. The expression levels of each gene were normalized to the mean of the no glaucoma samples. Gene expression levels after crush were from the microarray data and the expression levels of the crushed eyes were normalized to the contralateral controls. Results were presented as mean with 90% lower and upper confidence bounds. Many genes showed similar responses after these two injuries. The genes that had different responses were highlighted with black frames.

Similar to the reactive gliosis after optic nerve crush, glaucomatous ONHs also up-regulated the inflammation and cell proliferation genes (Fig. 12A), that suggested shared common mechanisms in these two injuries. However, many of the membrane channels and cell adhesion molecules responded very differently. For example, *Aqp4*, astrotactin 1 (*Astn1*), contactin 1 (*Cntn1*), and gap junction protein alpha 1 (*Gja1*) were down-regulated after optic nerve crush, but were up-regulated in glaucoma. This suggested that the remodeling of the astrocytes at the ONH may be quite different in different injury models. We also checked the intermediate filament proteins that were traditionally used as astrocyte markers (*Gfap*, nestin, and vimentin) and a newly discovered astrocyte marker (integrin beta 5). The only difference between the two models was that vimentin was increased in glaucoma but showed no change after nerve crush. Thus, we observed both similarities and differences in molecule profiles of reactive gliosis in the ONH in two different injury models.

## Discussion

In our study, we had two main questions: first, what is the time course of molecular events after a traumatic injury to the optic nerve that leads to axon degeneration and glial reactivity, and second, do the astrocytes (and other glial cells) return to a resting stage after injury or do they remain in a “reactive” state?

The transcriptome of the ONH after nerve crush showed profound changes, especially shortly after injury. Over 1300 genes were differentially expressed one day after injury. The numbers of differentially regulated genes then dropped continuously over the time of our study. Clustering of the differentially regulated genes revealed that the changes pass over the ONH in waves. The first wave (Groups A and B in Figure 6) consists of genes that are involved in inflammation and immune regulation. For example, the chemokines *Ccl2*, *Ccl3*, and *Ccl5* were prominently upregulated (Group A). These chemokines can be produced by activated astrocytes [31], and are expected to attract multiple leukocyte subtypes [32], [33]. Also upregulated were the proinflammatory cytokines *IL1b* and *IL6*. Group B also contained genes that reacted within one day, but stayed elevated for longer time than the genes in group A. Amongst the pathways identified in this group was Fc gamma receptor mediated phagocytosis. Astrocytes have been described to be phagocytically active both *in vitro* and *in vivo* [34], [35], [36]; however, the low-affinity Fc Receptors IIb and III, upregulated early in our study, were not found to be expressed in purified astrocytes in a recent study [20]. Therefore, it is likely that the upregulation of this pathway reflects the increased phagocytotic activity of microglia. As a component of the innate immune system, Toll-like receptors (TLRs), play a role in inflammation of the central nervous system [37]. A total of 10 TLRs have been identified in mice, and microglia express the full complement of them. Astrocytes, too, can express TLRs, though their repertoire is more restricted [38]. In our study, we found TLR 1, 4, 6, 7, and 8 to be upregulated after optic nerve crush. They reacted early (Groups A and B) and had returned to baseline by three weeks. Of these, astrocytes can express at least TLR4 [39]. In addition to these genes, we found evidence of tissue remodeling that started by the first day after optic nerve crush. Several matrix metallopeptidases were upregulated at that time point, such as *Mmp9*, *Mmp10*, *Mmp12*, and *Mmp13*. *Mmp9* is secreted in astrocytes and neurons, and is upregulated upon stimulation of TLR4 [40].

Partially overlapping in expression pattern with Groups A and B, we found a cluster of genes that was upregulated one day after injury and returned to baseline with every consecutive time point and had returned to baseline by 3 weeks. Prominent in this Group C were genes involved in DNA replication and cell cycle. With a short delay, a second wave of gene upregulation happened in the optic nerve that peaked 3 days after crush, and returned to baseline by 3 weeks (Group D). In this group, cell cycle genes were highly represented. Interestingly, in a model of ocular hypertension genes involved in cell cycle and proliferation were also found to be upregulated [17]. This indicates that cell proliferation plays a role in the reactive changes that happen in the optic nerve after various kinds of injury. We labeled the proliferating cells with BrdU, and found that they were positive for IBA1 staining. As IBA1 does not distinguish between microglia and macrophages that invade the optic nerve from the bloodstream, the proliferating cells could be of either type. Also in Group C was tenascin C, an extracellular glycoprotein that has been implicated in reactive astrocytosis. This gene was expressed in reactive astrocytes in our analysis (Figure 11). Tenascin C was also found to be upregulated in optic nerves of patients with open-angle glaucoma and in a rat ocular hypertension model, suggesting that tenascin C expression in the optic nerve may be an useful marker of glaucomatous damage [17], [41].

A third wave of gene expression changes was identified that peaked one week after injury (Groups E and F), and returned to baseline by 3 weeks (Group E) or stayed moderately elevated (Group F). The pathways differentially regulated in these groups were indicative of tissue remodeling and clearance of debris. For example, genes involved in lysosomal pathways, such as cathepsin B and E, acid phosphatase 5, and lysosomal acid lipase A were upregulated. Ongoing tissue remodeling was evidenced by the upregulation of genes involved in extracellular matrix-receptor interaction.

The fourth wave of differentially regulated genes (Group G) comprised genes that were either at baseline or downregulated one day after crush, and increased in expression through the third week. Although functional annotation did not identify enriched pathways in this group, genes in this group are of potential interest because their upregulation coincides with the morphological normalization of optic nerve astrocytes >14 days after injury [6]. Two transcription factors, *Klf5* and *Tfcp2l1*, are representatives of this group. Klf5 is one of the kruppel-like transcription factors expressed in embryonic stem cells and is involved in early embryogenesis. Recently, it has been found to be expressed in granular and pyramidal cells of the hippocampus [42] and retinal ganglion cells [43], and in astrocytes after ischemic insult to the brain [7]. *Tfcp2l1* (transcription factor cp2-like 1) is required for the formation of ephithelial ducts [44], and was expressed by cultured and mature astrocytes, but not neurons or oligodendrocytes [20]. The possible involvement of these transcription factors in astrocyte recovery after injury is a topic for further study.

Lastly, Group H contained genes that were consistently downregulated after optic nerve crush. This group contained several oligodendrocyte-specific genes, such as myelin basic protein, oligodendrocyte transcription factors 1 and 2, and myelin-associated glycoprotein. Pathway analysis showed that some metabolic pathways (drug metabolism and steroid biosynthesis) are in this group. Examples for these genes were glutathione S-transferase kappa 1, mu 7, and theta 1, alcohol dehydrogenase 1, Cyp2d22, Cyp51, and 24-dehydrocholesterol reductase. In addition to these, though not associated to any specific pathway, several members of the solute carrier family were amongst these downregulated genes.

Three months after injury, the number of differentially expressed genes dropped to 127. Unsurprisingly, oligodendrocye marker genes like myelin basic protein, oligodendrocyte transcription factor 1, and myelin-associated glycoprotein remained downregulated, as did genes that presumably stem from axonal RNAs from ganglion cells, such as kinesin 2 and neurofilament 3. The transcription factors *Klf5* and *Tfcp2l1* were still upregulated at that time.

The ONH is of particular interest in glaucoma, as it is believed to be the initial site of damage to the retinal ganglion cells in that disorder [11], [12], [15], [45], [46]. Studies of gene expression in models of ocular hypertension, human glaucoma, and an inherited model of glaucoma in mice have been carried out [17], [18], [29], [47]. Similar to what we observed after the optic nerve crush injury, genes involved in inflammation, cell proliferation and tissue remodeling are also upregulated in these experimental models of glaucoma.

It is noteworthy that there are differences in gene expression between different injury models and between injury to the brain parenchyma and the optic nerve. A recent study compared genomic analysis of mouse brain astrocytes after ischemic injury and LPS-mediated neuroinflammation and found that at least half of the observed changes differed between the two models [7]. We compared the lists of the 50 most upregulated genes under either of these two conditions with the genes differentially regulated in our model. In the case of ischemic injury, amongst those 50 genes, 14 were also found to be upregulated after optic nerve crush. For LPS-induced astrocyte reactivity compared to optic nerve crush, the number was only 6. In the optic nerve head, too, different types of injury affect different sets of genes. When restricting the comparison to mouse ONH, only 549 out of the 2056 gene probe sets that were differentially expressed after optic nerve crush were also differentially regulated in glaucomatous eyes [18]. This indicates that astrocyte reactivity is a heterogeneous process that depends on the nature of the injury and on the type of astrocyte.

Our results indicate that not only the type of injury but the time after injury will be important for therapeutic intervention. If the goal is acute modulation of the glial response, as in attempts to prevent possibly deleterious effects of the glial reaction, the initial window of opportunity may be the first three days, when blocking of chemokine receptors should prevent the influx of potentially harmful inflammatory cells. Such manipulations would have little likely benefit at later times. Conversely, a later attempt to reverse glial scarring would require recognizing that the glia in the weeks or months after a traumatic injury has returned to something approximating a resting state. Our findings are of course limited to glia of the optic nerve, but it is likely that analogous temporal shifts are present in other white matter tracts.

## Supporting Information

Table S1 **Quality control of RNA preparations and microarrays.** For each individual sample, the RNA concentration, yield, and RNA integrity number (RIN) is given. The median intensity for each individual array, the percentage of genes called present, and the percentages of array outliers and individual outliers are also listed.
(DOC)
