# Supplementary material for: The Time Course of Gene Expression during Reactive Gliosis in the Optic Nerve
Source: PLoS One. 2013 Jun 27;8(6):e67094. doi: 10.1371/journal.pone.0067094 (PMC3694957; doi:10.1371/journal.pone.0067094)
Supplement: Table S1 — Quality control of RNA preparations and microarrays. For each individual sample, the RNA concentration, yield, and RNA integrity number (RIN) is given. The median intensity for each individual array, the percentage of genes called present, and the percentages of array outliers and individual outliers are also listed. (DOC) [file pone.0067094.s001.doc]

| **Sample #** | **Sample Name** | **Tissue Type** | **RNA Conc. (ng/ul)** | **RNA Yield (ng)** | **RIN** | **Median Intensity*** | **P call %** | **% Array outlier** | **% Single outlier** |
| --- | --- | --- | --- | --- | --- | --- | --- | --- | --- |
| 1 | C2 | naïve control | 5.08 | 71.15 | 10 | 308 | 74.5 | 0.564 | 0.106 |
| 2 | C3 | naïve control | 4.26 | 59.67 | 9.7 | 270 | 73.9 | 0.141 | 0.036 |
| 3 | C5 | naïve control | 7.05 | 98.70 | 10 | 297 | 74.1 | 0.093 | 0.071 |
| 4 | C6 | naïve control | 5.05 | 70.73 | 9.7 | 241 | 73.3 | 1.697 | 0.136 |
| 5 | C7 | naïve control | 4.41 | 61.77 | 9.4 | 269 | 71.1 | 0.278 | 0.076 |
| 6 | 1D1R | 1 day - control | 7.26 | 101.58 | 9.9 | 276 | 74.2 | 0.071 | 0.038 |
| 7 | 1D2R | 1 day - control | 4.83 | 67.62 | 9.9 | 309 | 74.1 | 0.062 | 0.027 |
| 8 | 1D3R | 1 day - control | 4.21 | 58.94 | 10 | 291 | 73.6 | 0.115 | 0.042 |
| 9 | 1D4R | 1 day - control | 7.40 | 103.57 | 10 | 313 | 73.5 | 0.079 | 0.041 |
| 10 | 1D5R | 1 day - control | 4.90 | 68.60 | 10 | 313 | 73.8 | 0.101 | 0.028 |
| 11 | 1D1L | 1 day - crushed | 5.12 | 71.71 | 10 | 298 | 73.5 | 0.591 | 0.114 |
| 12 | 1D2L | 1 day - crushed | 3.08 | 43.12 | 9.8 | 303 | 73.3 | 1.234 | 0.109 |
| 13 | 1D3L | 1 day - crushed | 6.59 | 92.26 | 9.6 | 331 | 78.8 | 0.996 | 0.116 |
| 14 | 1D4L | 1 day - crushed | 3.00 | 41.94 | 9.6 | 270 | 74 | 0.489 | 0.072 |
| 15 | 1D5L | 1 day - crushed | 2.76 | 38.61 | 9.2 | 308 | 73.6 | 1.785 | 0.368 |
| 16 | 3D2R | 3 days - control | 3.24 | 45.42 | 10 | 317 | 72.9 | 0.19 | 0.064 |
| 17 | 3D3R | 3 days - control | 6.24 | 87.30 | 10 | 376 | 79.1 | 0.185 | 0.061 |
| 18 | 3D4R | 3 days - control | 2.42 | 33.82 | 10 | 393 | 78.8 | 0.331 | 0.073 |
| 19 | 3D5R | 3 days - control | 7.80 | 109.20 | 9.9 | 347 | 77.8 | 0.3 | 0.093 |
| 20 | 3D6R | 3 days - control | 3.67 | 51.38 | 9.8 | 418 | 77.8 | 0.141 | 0.053 |
| 21 | 3D2L | 3 days - crushed | 3.33 | 46.59 | 10 | 319 | 78.5 | 2.138 | 0.143 |
| 22 | 3D3L | 3 days - crushed | 15.49 | 216.86 | 10 | 470 | 81 | 0.78 | 0.066 |
| 23 | 3D4L | 3 days - crushed | 13.23 | 185.19 | 10 | 361 | 79.7 | 0.45 | 0.072 |
| 24 | 3D5L | 3 days - crushed | 10.18 | 142.46 | 10 | 379 | 79.5 | 0.375 | 0.077 |
| 25 | 3D6L | 3 days - crushed | 9.18 | 128.55 | 10 | 311 | 77.1 | 5.848 | 0.198 |
| 26 | 1W1R | 1 week - control | 6.23 | 87.16 | 9.3 | 270 | 72.6 | 0.326 | 0.178 |
| 27 | 1W2R | 1 week - control | 8.34 | 116.70 | 9.9 | 387 | 78.6 | 0.176 | 0.085 |
| 28 | 1W3R | 1 week - control | 2.85 | 39.96 | 9.3 | 332 | 79 | 1.375 | 0.195 |
| 29 | 1W5R | 1 week - control | 8.22 | 115.14 | 9.2 | 456 | 79.6 | 1.159 | 0.081 |
| 30 | 1W6R | 1 week - control | 6.45 | 90.24 | 9.8 | 304 | 77.3 | 0.172 | 0.058 |
| 31 | 1W1L | 1 week - crushed | 16.09 | 225.32 | 9.9 | 365 | 78.4 | 0.586 | 0.135 |
| 32 | 1W2L | 1 week - crushed | 10.10 | 141.34 | 9.9 | 338 | 77.8 | 0.789 | 0.082 |
| 33 | 1W3L | 1 week - crushed | 19.82 | 277.45 | 9.5 | 389 | 79.6 | 1.419 | 0.083 |
| 34 | 1W5L | 1 week - crushed | 16.44 | 230.22 | 9.9 | 322 | 79 | 1.194 | 0.114 |
| 35 | 1W6L | 1 week - crushed | 20.96 | 293.38 | 9.8 | 381 | 79.8 | 0.82 | 0.089 |
| 36 | 3W1R | 3 weeks - control | 6.99 | 97.83 | 9.6 | 397 | 77.1 | 0.212 | 0.052 |
| 37 | 3W3R | 3 weeks - control | 3.38 | 47.38 | 9.5 | 275 | 76.3 | 0.41 | 0.091 |
| 38 | 3W4R | 3 weeks - control | 3.75 | 52.53 | 9.0 | 260 | 76.7 | 0.595 | 0.102 |
| 39 | 3W5R | 3 weeks - control | 2.49 | 34.92 | 9.7 | 231 | 74.4 | 0.952 | 0.178 |
| 40 | 3W6R | 3 weeks - control | 5.64 | 78.93 | 9.6 | 233 | 76.3 | 0.428 | 0.107 |
| 41 | 3W1L | 3 weeks - crushed | 5.74 | 80.33 | 9.4 | 334 | 77.7 | 0.754 | 0.075 |
| 42 | 3W3L | 3 weeks - crushed | 5.90 | 82.60 | 9.3 | 257 | 75.5 | 0.745 | 0.111 |
| 43 | 3W4L | 3 weeks - crushed | 10.22 | 143.05 | 9.5 | 269 | 77.9 | 0.934 | 0.119 |
| 44 | 3W5L | 3 weeks - crushed | 6.94 | 97.10 | 9.6 | 229 | 75.5 | 1.133 | 0.144 |
| 45 | 3W6L | 3 weeks - crushed | 12.32 | 172.42 | 9.8 | 168 | 74.3 | 2.212 | 0.295 |
| 46 | 3M1R | 3 months - control | 4.55 | 63.70 | 9.0 | 290 | 80.6 | 1.922 | 0.175 |
| 47 | 3M2R | 3 months - control | 2.38 | 33.32 | 9.0 | 270 | 80.4 | 0.463 | 0.04 |
| 48 | 3M3R | 3 months - control | 2.77 | 38.75 | 9.0 | 398 | 80.3 | 0.264 | 0.024 |
| 49 | 3M4R | 3 months - control | 3.20 | 44.80 | 9.0 | 363 | 79 | 0.19 | 0.11 |
| 50 | 3M5R | 3 months - control | 2.55 | 35.67 | 9.3 | 349 | 78.5 | 0.123 | 0.019 |
| 51 | 3M1L | 3 months - crushed | 3.67 | 51.35 | 9.0 | 311 | 80.7 | 0.181 | 0.031 |
| 52 | 3M2L | 3 months - crushed | 4.34 | 60.76 | 9.5 | 282 | 76.3 | 0.234 | 0.032 |
| 53 | 3M3L | 3 months - crushed | 4.08 | 57.12 | 9.3 | 271 | 76.8 | 0.472 | 0.044 |
| 54 | 3M4L | 3 months - crushed | 7.78 | 108.92 | 9.6 | 277 | 79 | 0.309 | 0.083 |
| 55 | 3M5L | 3 months - crushed | 6.27 | 87.81 | 9.5 | 318 | 80 | 0.304 | 0.021 |

* Median Intensity before normalization.

Table S1. Quality control of RNA preparations and microarrays. For each individual sample, the RNA concentration, yield, and RNA integrity number (RIN) is given. The median intensity for each individual array, the percentage of genes called present, and the percentages of array outliers and individual outliers are also listed.
